# Harter’s Iron Tonic

**Published:** 1882-04-20

**Authors:** 


HARTER'S IRON TONIC.—Read the advertissment of this medicine. The Formula stikes us most favorably. It is evidently a fine combination.

